# The Role of Sensory Feedback in Developmental Stuttering: A Review

**DOI:** 10.1162/nol_a_00036

**Published:** 2021-06-09

**Authors:** Abigail R. Bradshaw, Daniel R. Lametti, Carolyn McGettigan

**Affiliations:** Department of Speech, Hearing & Phonetic Sciences, University College London, UK; Department of Psychology, Acadia University, Canada

**Keywords:** developmental stuttering, speech motor control, altered feedback paradigm, sensorimotor integration, sensory feedback

## Abstract

Developmental stuttering is a neurodevelopmental disorder that severely affects speech fluency. Multiple lines of evidence point to a role of sensory feedback in the disorder; this has led to a number of theories proposing different disruptions to the use of sensory feedback during speech motor control in people who stutter. The purpose of this review was to bring together evidence from studies using altered auditory feedback paradigms with people who stutter, in order to evaluate the predictions of these different theories. This review highlights converging evidence for particular patterns of differences in the responses of people who stutter to feedback perturbations. The implications for hypotheses on the nature of the disruption to sensorimotor control of speech in the disorder are discussed, with reference to neurocomputational models of speech control (predominantly, the DIVA model; Guenther et al., 2006; Tourville et al., 2008). While some consistent patterns are emerging from this evidence, it is clear that more work in this area is needed with developmental samples in particular, in order to tease apart differences related to symptom onset from those related to compensatory strategies that develop with experience of stuttering.

## INTRODUCTION

Sensory feedback processing is known to play a crucial role in speech production, and forms a central part of many models of speech motor control (Guenther et al., 2006; Hickok et al., 2011; Parrell, Ramanarayanan, et al., 2019). Auditory feedback is thought to play a particularly important role in learning to produce speech sounds early in development, as well as continuing to guide and maintain accurate speech production throughout the lifetime (Guenther, 2016). For example, not only do congenitally deaf individuals struggle to develop typical speech production (Smith, 1975; Svirsky et al., 2004), but those who lose their hearing as adults can also show gradual changes in articulation over time (Cowie & Douglas-Cowie, 1992). Disruptions to somatosensory feedback have been shown to have even more profound effects on speech production in the short term, with temporary oral anaesthesia resulting in highly inaccurate articulation (Ringel & Steer, 1963; Scott & Ringel, 1971). Such evidence has led researchers to propose that the internal targets that guide speech motor control are sensory in nature, and to stress the importance of feedback loops that compare the intended/predicted and actual sensory consequences of a speech production (Guenther et al., 2006; Parrell & Houde, 2019).

### Sensory Feedback and Developmental Stuttering

Developmental stuttering is a disorder that involves impairment in speech fluency. The speech of people who stutter (PWS) is characterised by frequent repetitions and prolongations of syllables, as well as tense pauses in which a speech sequence fails to be initiated (known as *blocks*). The onset of the disorder typically occurs in early childhood, with a prevalence of up to 8% in pre-school children (Yairi & Ambrose, 2013); however, the majority (around 80%) spontaneously recover during childhood, resulting in an incidence of chronic lifetime stuttering of around 1% in the general population (Craig et al., 2002). Stuttering has been studied at multiple levels, from genes to behaviour, yet the causes of stuttering remain unknown. One prominent account of the speech motor control aspects of the disorder proposes that stuttering involves disruption to sensorimotor integration. The most overt evidence for this comes from observations that dramatic increases in the fluency of PWS can be achieved temporarily by altering auditory feedback during speech (for a review, see Lincoln et al., 2006). In these studies, PWS speak into a microphone and their speech is played back to them in real-time over headphones. Alterations of the feedback—typically, delays, frequency shifts, or masking—can reduce stuttering frequency by up to 90% (Bloodstein & Ratner, 2008; Foundas et al., 2013; Kalinowski et al., 1993). However, these fluency-enhancing effects are temporary; effects can start to wear off as the speaker “adapts” to the particular feedback alteration, and they do not persist once feedback is returned to normal.

Such observations have led to great interest in the role of sensory feedback in stuttering. A number of authors have proposed that disruption in the use of auditory feedback during speech motor control may contribute to speech dysfluencies (e.g., Max et al., 2004). There has also been some interest in the idea that somatosensory feedback processing may be disrupted in PWS (Archibald & De Nil, 1999; Loucks & De Nil, 2006). Neural evidence further points to a possible disruption in sensorimotor integration, with reports of altered structure and function in multiple relevant brain areas in PWS, such as the left inferior frontal gyrus (IFG), primary motor cortex (PMC), and posterior superior temporal gyrus STG; (Brown et al., 2005; Watkins et al., 2008; for a review, see Chang et al., 2019).

However, the precise nature of the disruption to sensory feedback processing in PWS remains unknown. Sensory feedback has multiple roles to play in speech production, from calibration of stored sensory targets to the guidance of online adjustments to an unfolding motor programme (Parrell & Houde, 2019). Accordingly, multiple theoretical perspectives have been suggested that propose different sites of disruption to auditory feedback processing in stuttering (Chang & Guenther, 2020; Max & Daliri, 2019; Max et al., 2004). These make contrasting predictions as to the behaviour of PWS in situations in which sensorimotor integration is required.

### The Altered Feedback Paradigm

**Figure 1.**
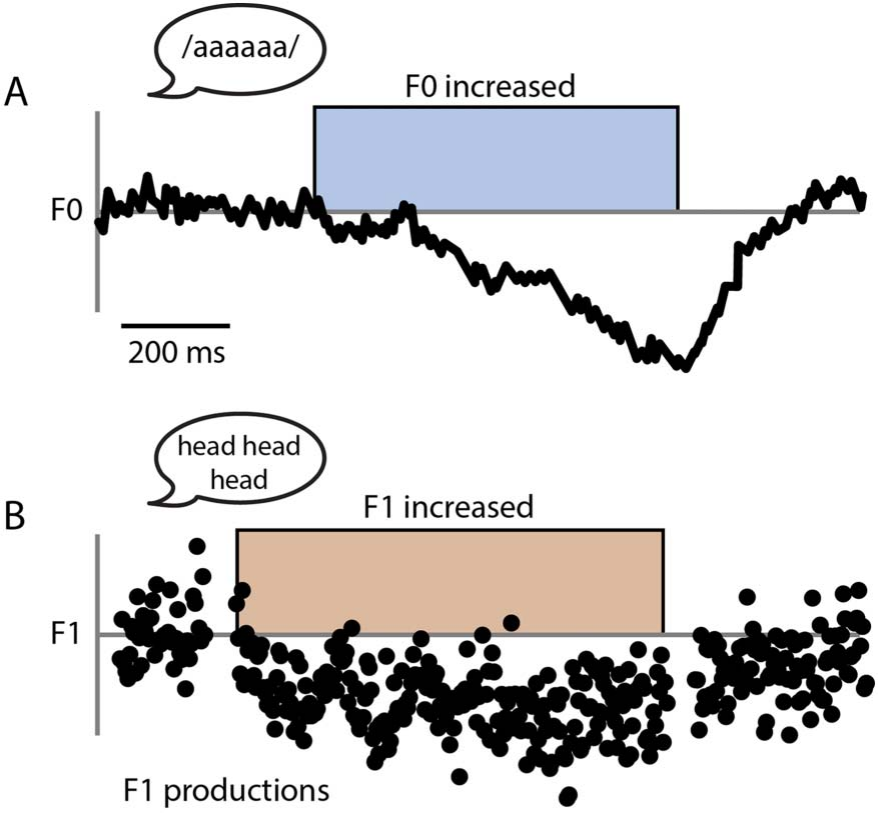
Unexpected and sustained perturbation paradigms. (A) Unexpected perturbation paradigms typically require participants to sustain phonation of vowel sounds for a duration of several seconds. On a proportion of trials, feedback perturbations are unexpectedly applied with random onset during vocalisation, typically with both upward and downward shift trials (upwards F0 perturbation pictured). Speakers typically display reactive changes to the current vocalisation so as to oppose this perturbation during the trial, termed *the compensation response*. (B) Sustained perturbation paradigms typically require participants to repeat words (with normal vocalisation timing) across many trials. After a baseline period with normal feedback, a feedback perturbation is then applied in a consistent fashion across trials (upwards F1 perturbation pictured). Across many trials, the speaker typically starts to gradually change their productions so as to oppose the perturbation, termed *the adaptation response*. Feedback is typically then returned to normal for a final block of trials, to look at after-effects of adaptation. Adapted from Burnett et al. (1998) and Lametti et al. (2018).

One major paradigm for investigating auditory-motor integration during speech is the altered feedback paradigm (Houde & Jordan, 1998). This involves perturbation of the auditory speech feedback a speaker hears in real time, typically in the form of a shift in fundamental frequency (F0) or formant frequencies. These feedback perturbations can be either unexpected, with upward and downward shifts applied randomly to utterances (Burnett et al., 1998); or sustained, such that the same type and level of perturbation is consistently applied across multiple utterances (Houde & Jordan, 1998). In both unexpected and sustained cases, participants are found to demonstrate compensatory adjustments to their speech productions so as to oppose the effects of the perturbation (see Figure 1 for more details). This has the effect of moving the auditory feedback from utterances closer to baseline (pre-perturbation) levels. Crucially, the responses induced by unexpected and sustained perturbations of auditory feedback are proposed to reflect the operation of different underlying subsystems within speech motor control (see section, Feedforward versus Feedback control). These paradigms therefore offer much potential for teasing apart the nature of the possible deficits in auditory-motor integration in PWS.

## AIMS OF THE CURRENT REVIEW

Over the last decade, there has been a growing number of studies using such altered feedback paradigms with samples of PWS. Despite the prominence of the idea of disrupted sensorimotor integration in stuttering, there has been no previous effort to bring together this evidence in order to explicitly test and evaluate the predictions made by different theories. The current paper therefore aims to provide the first review of studies using altered feedback paradigms with PWS, with specific reference to predictions made by different theories of speech motor disruption in stuttering. By reviewing this evidence, we aimed to identify if PWS show consistent differences in their responses to altered auditory feedback that may reveal how sensory feedback processing is disrupted in the disorder.

This review will begin by summarising the key theories that propose a disruption to the use of auditory feedback in speech motor control in PWS. This will highlight the similarities and differences between theories, and their predictions for responses of PWS in altered feedback experiments. We will then review evidence from studies using the altered feedback paradigm with PWS with reference to these predictions, in order to evaluate which theory is best supported by the evidence. In so doing, we will identify key phenomena that must be explained by such theories, as well as areas that require further theory development.

### Introduction to Speech Motor Control: The DIVA Model

Before proceeding to summarise key theories of feedback processing disruption in stuttering, it is useful to provide a brief introduction to core concepts and terminology used within the field of speech (and indeed non-speech) motor control.

Multiple formal models of speech motor control have been presented which, although drawing on many of the same concepts, have distinct functional architectures. These include the Directions into the Velocities of Articulators (DIVA) model (Guenther et al., 2006; Tourville et al., 2008); the Task Dynamics framework (Saltzman & Kelso, 1987; Saltzman & Munhall, 1989); State Feedback Control (SFC) (Hickok et al., 2011; Houde & Nagarajan, 2011); and the Feedback Aware Control of Tasks in Speech (FACTS) model (Parrell, Ramanarayanan, et al., 2019). A recent comparative review of these different speech motor control model architectures was led by Parrell, Lammert, et al. (2019). The current review, however, will focus on the DIVA model, because this is the only framework that has been applied to stuttering; indeed, the majority of the theories and studies reviewed in the current paper draw directly on the DIVA model. To aid understanding throughout discussion of such research in the following sections, we will therefore begin with a brief outline of some core concepts within computational models of speech motor control, and how these are implemented in the DIVA model.

#### Feedforward versus Feedback control

**Figure 2.**
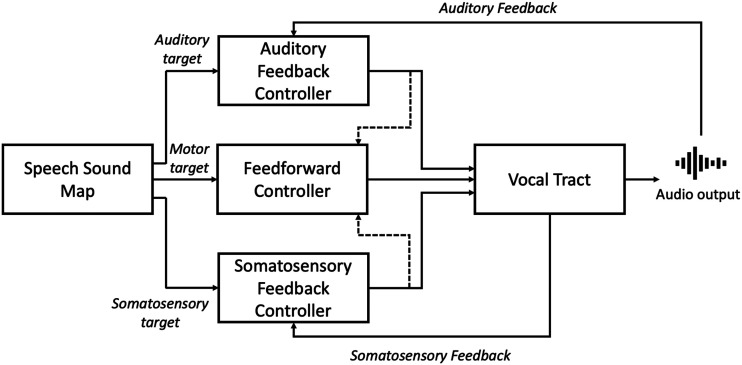
Simplified schematic of the DIVA model control system. Motor commands sent to the vocal tract are the result of the summed output of the three control subsystems: the Feedforward controller, the Auditory Feedback controller, and the Somatosensory Feedback controller. The feedback controllers compare sensory feedback to their corresponding sensory targets for a speech production, and if needed issue online corrective motor commands based on detected errors. Dashed arrows indicate the use of these corrective commands to update the feedforward commands offline. Adapted from Guenther (2016).

At the centre of computational approaches to motor control is a primary distinction between two types of control systems: feedback control and feedforward control. These two systems use sensory feedback in different ways. The *feedback control* system uses sensory feedback for online control of movements. During speech production, the sensory consequences of an articulatory gesture are compared to the predicted/desired sensory outcome; any discrepancy between these generates an error signal that is used to correct the ongoing movement trajectory online. In contrast, a purely *feedforward control* system issues pre-planned motor commands that are not corrected online; sensory feedback is instead used to train and update these feedforward commands offline, so that future movements are performed accurately from the outset. The DIVA model combines these two control systems, with the feedback controller being further subdivided into two modality specific controllers, one for somatosensory feedback and one for auditory feedback. There are thus three key components of speech motor control in DIVA: the feedforward controller, the auditory feedback controller, and the somatosensory feedback controller. Speech motor commands are generated by summing the outputs of these three controllers. This control system is illustrated in Figure 2.

According to DIVA, speech motor control early in speech development relies exclusively on feedback control, with motor commands being generated online using sensory (predominantly auditory) feedback. Over time, the output from the feedback controller is used to train the feedforward controller, so that accurate feedforward commands can be learnt. This corresponds to the formation of *speech sound map nodes*, which encode the motor, auditory, and somatosensory target trajectories associated with each speech sound. Once these become sufficiently reliable, there is a shift in balance between the systems, such that the feedforward system takes over as the dominant form of speech motor control. However, the presence of the feedback controller means that speech motor control remains sensitive to errors in sensory feedback. The DIVA model can thus explain the ability of speakers to compensate for artificially induced perturbations of speech feedback during altered feedback experiments.

Feedback and feedforward modes of motor control have different associated benefits and limitations. A feedback control system can allow for sensitivity to changes in auditory feedback, and as such is critical for supporting the initial learning of speech sounds as outlined above. A speech motor control system that continued to rely purely on feedback control, however, would be severely limited in the range of movement speeds it could handle; processing of sensory feedback involves delays of up to 150 ms, prohibiting rapid speech movements. These delays can also result in greater instability in motor control, since feedback-based corrections to ongoing movements are likely to be triggered too late in the speech sequence, leading to overshoots and potentially oscillatory behaviour. In contrast, the feedforward system can generate motor commands for an utterance before sensory feedback from that production is available, enabling the production of fast and precise articulatory movements that characterise much of our connected speech. The feedforward system, however, relies on the feedback system to detect any sensory errors in produced speech, in order for stored feedforward commands to be updated so as to reduce errors in future utterances. In this way, a control scheme that combines these two controllers can balance their relative advantages and disadvantages.

Importantly, the two major types of altered feedback paradigm (unexpected versus sustained) are proposed to differentially engage the two control systems. Unexpected perturbations are thought to engage the feedback control system, which supports the online correction of sensory errors during production of the perturbed utterance (hereafter termed *compensation responses*). Conversely, sustained perturbations are proposed to induce sensorimotor learning via the feedforward system, in which there is a gradual updating of stored motor plans based on the consistent errors in sensory feedback (hereafter termed *adaptation responses*). Evidence that such changes in stored feedforward commands have occurred is found in the existence of aftereffects of adaptation, in which changes to speech persist for a time after the perturbation has been removed, with a gradual “wash-out” and return to baseline levels over time (Purcell & Munhall, 2006). As highlighted above, however, this updating of feedforward commands relies on the detection of consistent errors in speech feedback by the feedback control system; thus, adaptation responses in sustained perturbation paradigms reflect the joint operation of the feedback and feedforward control systems. Some experiments have attempted to isolate the contribution of the feedforward system during sustained perturbations, such as including noise-masked trials in which feedback control is not possible (Houde & Jordan, 1998), or restricting analysis to an early time-window within each utterance before feedback-based corrections can be initiated (Parrell et al., 2017). Keeping this point in mind, it can still be seen that contrasting these two perturbation paradigms can allow for comparisons of the operation of the feedback versus feedforward control systems.

#### Internal models in speech motor control

Both the feedback and feedforward control systems make use of what are termed *internal models.* These act to translate between motor commands and their associated sensory outcomes. Two types of internal models are described in the speech motor control literature: *forward models*, which translate motor commands into predicted sensory consequences; and *inverse models*, which translate sensory outcomes into motor commands. Inverse models are used by the feedforward system to enable transformation of a desired sensory goal into the motor commands needed to achieve that goal. The feedback system uses forward models to allow a comparison between incoming sensory feedback from a speech articulatory gesture and some kind of prediction of that feedback. The source of that prediction, however. varies between different theories. One long-standing idea is that the prediction is obtained from an “efference copy” of the motor commands (Wolpert et al., 1995); specifically, when motor commands are sent to the articulators, a prediction of their sensory consequences is simultaneously sent to sensory cortex via reafference, in order to allow a “subtraction” of incoming sensory input from this sensory prediction. This process of prediction generation through efference copy is used in the SFC and FACTS model frameworks, in order to help provide an internal estimate of the current state of the articulators (see Parrell & Houde, 2019, for a review). Comparison of this prediction with incoming sensory feedback can then be used to update this estimate. Although early versions of the DIVA model also used efference copy as a source of sensory predictions in forward models (Guenther, 1995; Guenther et al., 1998), the most recent versions propose that these predictions come from the sensory target/goal for the intended utterance. That is, the forward model allows a comparison between the *intended* and actual incoming sensory feedback. In DIVA, inverse models in the feedback system are then used to translate any detected sensory error into corrective motor commands. Again, however, it should be noted that this proposal is unique to DIVA; the motor control models from which these terms originated do not consider inverse models to be part of the feedback system or to receive any information on sensory errors (Wolpert et al., 1998). Overall, however, it can be seen that sensory feedback has a key role to play in establishing the sensory-motor mappings that underpin these internal models.

#### Neural basis of the DIVA model

**Figure 3.**
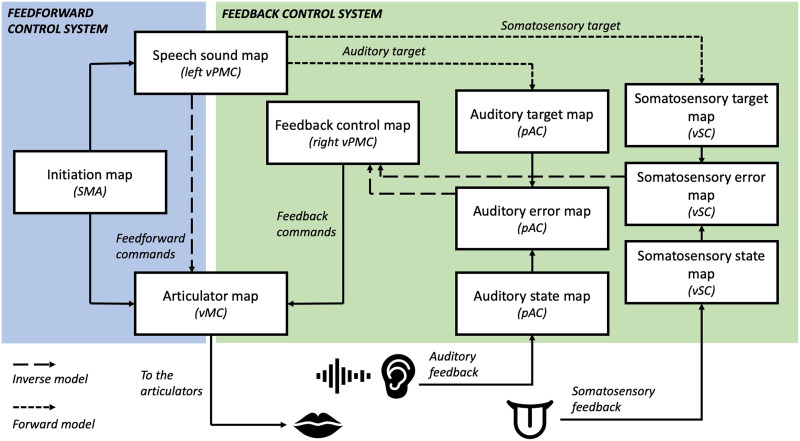
The DIVA model of speech motor control. Schematic shows different components involved in speech motor control and their hypothesised neural bases according to the DIVA model. Note that loops through subcortical structures are not shown on this diagram. Adapted from Guenther (2016).

A key feature of the DIVA model is that it is not only mathematically defined at the computational level, but also neurally specified. Thus, in the model, different components of the feedback and feedforward control systems have been attributed to different neural substrates. These are illustrated in Figure 3. Production of a speech sound begins with activation of a *speech sound map node* in the left ventral PMC; these are activated by *initiation nodes* in the supplementary motor area (SMA), in turn activated by either the pre-SMA or basal ganglia. Projections from the speech sound map to the articulator map in bilateral primary motor cortex then constitute the feedforward motor commands for a speech sound. In the feedback control system, projections from the speech sound map to posterior auditory cortex (pSTG) act as a forward model that predicts the intended sensory consequences of the target speech sound. Posterior auditory cortex then facilitates comparison of this sensory target with actual sensory feedback, which may result in activation of an auditory error map. Projections from posterior auditory cortex to a feedback control map in right vPMC translate this auditory error into corrective motor commands (via inverse models), which are sent on to the articulator map in motor cortex.

Across these various points of processing, the DIVA model proposes the use of both cortico-cortical and cortico-subcortico-cortical connections, via structures such as the basal ganglia and cerebellum. The DIVA model has also been extended to account for the planning, timing, and coordination of multisyllabic speech sequences; this is the Gradient Order DIVA (GODIVA) model (Bohland et al., 2010). This incorporates processing within a range of brain regions involved in working memory and motor sequencing, such as the posterior inferior frontal sulcus, vPMC, SMA, pre-SMA, and basal ganglia. Interestingly, brain imaging evidence from samples of PWS has reported abnormalities in many of the regions implicated in the DIVA and GODIVA models, including the left inferior frontal gyrus (IFG), the pSTG, and the basal ganglia (for a review, see Chang et al., 2019).

### Theories of Disruption to Feedback Processing in Stuttering

Multiple authors have suggested disruptions to the normal use of auditory feedback in speech motor control in stuttering, often based in disturbances of the relative balance of dominance between the feedforward and feedback control systems. These theories will be reviewed in the following sections, ending with a summary of their key predictions for responses to alterations of auditory feedback during speech production in PWS.

#### The *Speech Motor Skills* account

The *Speech Motor Skills* (*SMS*) view of stuttering proposes that PWS are at the lower end of a motor skill continuum (van Lieshout et al., 1996a, 1996b). In typical individuals, execution of movements gradually shifts from explicit to implicit control with practice, resulting in the movement becoming automated. Other hallmarks of skilled movement control include being energy efficient, having the ability to flexibly adapt to changing task requirements, and being able to optimise movements achieving specific goals. In contrast, limited motor skill would be reflected in error prone movements that are more variable, more slowly executed, less flexible, and that show limited improvement with practice. According to the *SMS* account of stuttering, limited speech motor skill in PWS thus results in speech movements that are less automatised and efficient (i.e., less implicit). This view is based upon a body of evidence reporting less proficient motor performance in PWS, across both speech and nonspeech tasks. In particular, PWS appear less able to benefit from practice during motor skill learning, as measured during finger tapping (Smits-Bandstra & De Nil, 2007), nonsense word learning (Namasivayam & van Lieshout, 2008), and syllable sequencing tasks (Smits-Bandstra et al., 2006).

In order to compensate for this reduction in motor skill, PWS are proposed to increase dependence on sensory feedback during speech motor control. For example, this can be achieved by slowing the rate of speech, something that has been reported in the fluent speech of PWS (Zimmermann, 1980) and is frequently employed in therapeutic interventions (Bloodstein & Ratner, 2008; Onslow & Ingham, 1987). Thus, according to this view, differences in the use of sensory feedback for speech motor control in stuttering are part of a compensatory strategy; that is, feedback control itself is not disrupted and can be used to help prevent stuttering. However, there are costs involved with this increased reliance on feedback control: Movements are more time-consuming, they place greater demands on attentional resources, and the range of movement speeds that can be dealt with effectively by the system is restricted (i.e., slower movements are favoured). Relating this to the DIVA model framework, this account can be conceptualised as a reduced reliance on feedforward planning and an increased reliance on feedback control.

#### Unstable or insufficiently activated internal models

In a seminal paper, Max et al. (2004) presented two hypotheses regarding potential causes of speech dysfluency in stuttering, based within the DIVA model framework. The first of these hypotheses proposed that stuttering involves disruption to the internal models that facilitate the transformation between motor commands and sensory consequences. In this account, stuttering is proposed to involve an impairment in the ability to use auditory feedback early in development to establish such bidirectional mappings between motor commands and sensory consequences. The authors suggest that this could be underpinned by a disruption to cerebellar or basal ganglia based learning.

Disruption to the learning, retention, and updating of both types of internal models will have multiple implications for speech motor control. Firstly, disrupted inverse models will result in inaccurate feedforward motor commands, increasing the need for feedback-based correction of errors. On top of this, disruption to forward models will result in inaccurate prediction of the expected sensory consequences of those commands within the feedback control system. In this way, there is both an increase in production errors, and simultaneously an impairment in the ability of the feedback system to anticipate and correct for such errors. Indeed, such faulty forward model predictions could result in error signals being erroneously generated, triggering a correction of otherwise correctly executed movements.

Ultimately, the system will be forced to rely more on a purely afferent feedback control strategy (i.e., reliant on actual sensory feedback without any forward modelling or prediction of that feedback). Such over-reliance on feedback control is associated with increased instability, due to delays inherent in feedback processing (see previous section, Feedforward versus Feedback control). Like the speech motor skills account, this theory thus proposes that slowing of speech can act as a compensatory strategy for PWS. Longer movement durations would allow the system to make better use of afferent feedback processing, in the face of faulty modelling of feedback. Therefore, slowed speech is again seen as a compensatory mechanism, rather than as a symptom of an underlying limitation.

A slight variation on these ideas was proposed by Hickok et al. (2011), in relation to their hierarchical state feedback control model of speech production. They similarly suggested that stuttering involves inaccurate forward predictions of the sensory consequences of a speech gesture. However, this was attributed to noisiness in the mapping between an internal estimate of the state of the vocal tract and the sensory system. This will result in inaccurate generation of error signals, as proposed above. They suggested that noisiness in this mapping was caused by disruption to area Spt (a region located at the parietal-temporal boundary in the left posterior Sylvian fissure).

It is worth highlighting here that while the DIVA model assumes the use of inverse models in the feedback system to translate detected sensory errors into corrective motor commands, this view is not shared by the *Unstable Internal Models* hypothesis; therefore, discussion of faulty inverse models in the feedback system is not included in this theory.

#### Overreliance on feedback control

The second hypothesis put forward by Max et al. (2004) proposed that stuttering involves weakened feedforward control that leads to an overreliance on feedback control. That is, early in childhood, stuttering individuals fail to show the normal transition from greater reliance on feedback control to increased reliance on the feedforward system for mature speech motor control. Unlike in the *SMS* account of stuttering, however, this increased reliance on sensory feedback is not considered to help compensate for stuttering, but instead is a cause of speech dysfluency. Specifically, the delays inherent in afferent feedback cause speech motor control to become unstable. Although similar to the first hypothesis presented by these authors (reviewed above), critically this hypothesis does not assume any disruption to internal models or to the feedback system itself. Instead, the core site of disruption is within feedforward control, forcing the system into an overreliance on feedback control of speech.

This theory has been further developed in subsequent papers by Guenther and colleagues. Civier et al. (2010) proposed that an overreliance on feedback leads to an accumulation of large sensorimotor errors, due to the time-delays associated with detection and correction of errors in the feedback system. These errors are considered to be present in the fluent speech of PWS; however, when they become sufficiently large, a hypothesised “monitoring subsystem” detects the excessive error. This triggers a motor reset in which the system attempts to repair the error by restarting the syllable, resulting in a repetition. According to this model, each repetition constitutes an unsuccessful production (i.e., a production with high error); corrective commands sent to the articulators should mean that each successive repetition involves progressively less auditory error. The intended utterance can then proceed (i.e., fluent speech continues) when the error has been reduced to a sufficiently low level.

Simulations within the DIVA and GODIVA models have been used to provide support for this account (Civier et al., 2010; Civier et al., 2013). Using the DIVA model, Civier et al. (2010) were able to replicate multiple features of speech in PWS by simulating reduced gain of feedforward and increased gain of feedback control. Specifically, this “stuttering DIVA” simulation produced repetitions of syllables, as well as auditory errors in fluent speech in the form of delayed formant transitions. This was especially seen on tokens that involved bilabial consonants followed by high-F2 vowels (e.g., /bid/). This latter finding replicates findings of slowed and delayed formant transitions in PWS, again typically on transitions involving high-F2 vowels (Chang et al., 2002; Robb & Blomgren, 1997). According to Civier et al. (2010), overreliance on the feedback system would be expected to have a particularly detrimental effect on production of such tokens, as they require the production of rapid transition rates that cannot be supported by the sluggish feedback system. Interestingly, simulations of slowed speech and masking of auditory feedback were found to reduce dysfluencies in the model. Further simulations by Civier et al. (2013) using the GODIVA model suggested that the nature of the disruption to the feedforward system may involve either a disconnection of cortico-striatal pathways or a dysregulation of the dopamine system. Both these simulated disruptions were found to result in stuttering behaviour due to delayed readout of the motor programme for the next syllable in a speech sequence.

Again, it should be stressed that in this account both the feedback control system and the sensitivity of PWS to sensorimotor errors is considered to be normal; the only site of impairment is in the read-out of feedforward commands, which causes a bias towards feedback-based control. However, even though the feedback system is functioning normally, it is itself the cause of speech errors due to its associated limitations, for example, in the range of movement speeds that it can handle. This is proposed to underlie the slowing of speech typically seen in PWS; that is, rather than being seen as an intentional compensatory strategy to aid fluency, slowness of speech is construed as an inevitable result of a dysfluency-inducing weakness (that is, overreliance on feedback control). As previously described, this limited speed of speech can itself then lead to increased sensorimotor errors, as some formant transitions do not occur at their target speed. The efficacy of masking auditory feedback in improving fluency is attributed to a reduced ability to detect small errors in sensory feedback, and thus a reduced likelihood of a repetition being triggered. In this way, any conditions that lead to a reduction in the detection of speech errors by the feedback control system would be predicted by this account to improve speech fluency.

#### Impaired left-hemisphere basal ganglia motor loop

Recently, Guenther and colleagues have proposed a variation on these ideas concerning the role of auditory feedback processing in stuttering (Chang & Guenther, 2020; Guenther, 2016). In this account, stuttering is rooted in disruption of the basal ganglia motor loop, thus building on previous models implicating this group of structures in the pathology underlying stuttering (Alm, 2004; Giraud et al., 2008). After considering a number of possible disturbances to basal ganglia functioning, Guenther and colleagues focus on an account that considers interactions between auditory feedback and the basal ganglia “initiation circuit.” This account draws on the GODIVA model’s proposed role of the basal ganglia in generating initiation and termination signals for moving between motor programmes representing different phonemes within a speech sequence. Crucially, this process is guided by monitoring of the current cognitive, motor, and sensory context; when the basal ganglia detect a match between the current context and that required by an upcoming speech gesture, they signal the SMA to initiate the next phoneme in the sequence.

According to this account, the core deficit in stuttering is in left hemisphere cortical areas involved in feedforward control, which results in small articulatory errors in speech. These errors affect the fluid initiation and termination of speech motor commands by the basal ganglia. Specifically, detection of these errors in auditory feedback means that the current sensory context does not match that required for initiating the next motor programme. The basal ganglia are consequently unable to move forward in the speech sequence, resulting in dysfluency. Therefore, although the core disruption in this account is in the feedforward system, it is processing within the feedback system that ultimately leads to breakdowns in speech fluency.

In this way, involvement of auditory feedback control mechanisms is considered by this account to be maladaptive and a major contributing factor to stuttering, not a compensatory strategy. Instead, it is proposed that PWS may in fact try to suppress processing of auditory feedback in order to prevent the detection of feedback errors that contribute to speech dysfluencies. This is based on findings of reduced activity in auditory cortical regions in PWS, identified by meta-analyses as one of the most common findings across imaging studies in stuttering (Brown et al., 2005; Budde et al., 2014). Therefore, in contrast to previous proposals related to overreliance on feedback, this account predicts a gradual reduction in reliance on feedback with experience of stuttering, as a learnt compensatory strategy. Fluency enhancing conditions that involve alterations of speech feedback (e.g., delaying, masking) are also proposed to reduce or prevent detection of articulatory errors, allowing speech sequences to unfold unimpeded.

#### Disrupted auditory prediction and feedback monitoring

A distinct view of the role of auditory feedback in stuttering was presented in a recent paper by Max and Daliri (2019). In contrast to previously reviewed accounts that tend to assume no disruption to the processing of feedback itself, this account argues for impaired auditory feedback processing in PWS. Specifically, they proposed that stuttering involves a disruption in the use of auditory prediction to prime the auditory system prior to onset of a speech movement. Such priming may optimise the tuning characteristics of auditory cortex, in order to prepare for processing of upcoming auditory feedback. A failure to do so may lead to aberrant feedback-driven corrective commands, triggering unnecessary repairs of speech movements to result in a breakdown of fluency during speech production. In this account, therefore, it is not the process of feedback monitoring that is affected *per se*, but processes involved in priming the auditory cortices prior to movement onset that have a knock-on effect for their upcoming role in sensory error detection.

This hypothesis was based on a series of studies reporting a lack of pre-speech auditory modulation (PSAM) in people who stutter (Daliri & Max, 2015a, 2015b, 2018). Specifically, in people who do *not* stutter (PWNS), the auditory evoked potential (AEP) measured in response to an auditory probe was significantly smaller when presented during a period of speech motor planning in a delayed-response speech task, than when presented at the same point in time in a silent reading condition. Conversely, PWS were found to lack this PSAM effect. This normal reduction of the AEP in PSAM was not interpreted as a general suppression of the auditory system, but rather an enhancement of its sensitivity to auditory feedback, and specifically, to errors in that feedback. Interestingly, the same pattern of results was found when investigating auditory modulation during a listening condition, in which a written word stimulus was followed by a recording of the participant’s voice speaking the word (Daliri & Max, 2015a). Again, PWNS showed significant modulation of the AEP in this listening condition relative to silent reading, whereas this modulation effect was absent in a group of PWS. They thus argued that reduced PSAM in PWS reflects a general disruption to prediction of upcoming auditory inputs, whether self- or externally-generated, rather than a disruption to motor command planning.

According to these authors, production of simple monosyllabic words in isolation is assumed to be reliant on feedforward control mechanisms; production of longer more complex multisyllabic utterances is by contrast considered to place greater demands on feedback monitoring and error correction, thus increasing the likelihood of a breakdown in fluency. This is in contrast to previously reviewed theories, which consider the feedback system to be restricted in its ability to handle faster movement speeds as in fluid connected speech utterances, thus benefiting from a slowing of speech utterances. The authors also propose that the mechanisms involved in PSAM may contribute more to online feedback-driven corrections, being less involved in longer-term speech adaptation and updating of stored internal models.

Overall, the functional relevance of PSAM is as yet unproven. Promising evidence for its contribution to feedback control, however, is suggested by the finding that PSAM is reduced in PWNS when speaking with predictable delays in auditory feedback (Daliri & Max, 2018); this is consistent with the idea that PSAM-related mechanisms for enhancing feedback monitoring may disengage when auditory feedback is rendered non-informative.

#### Comparison of theories and predictions

**Figure 4.**
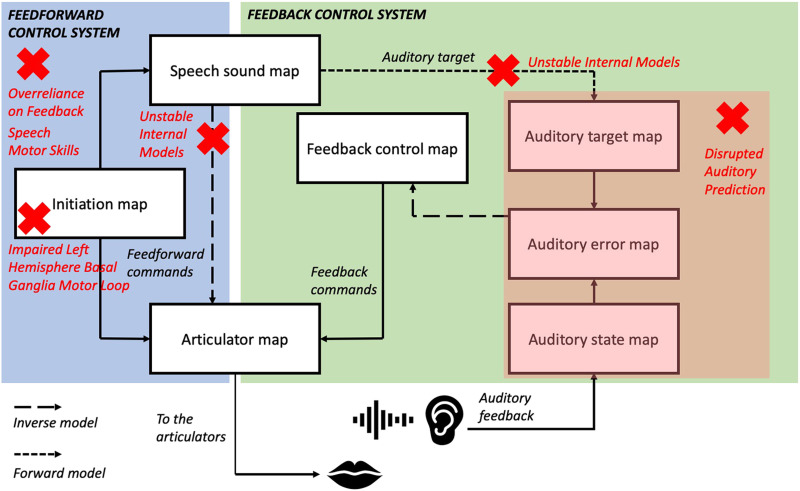
Comparison of sensorimotor accounts of stuttering within the DIVA model framework. Red crosses denote main areas of disruption proposed by different theories, within a simplified DIVA model framework. Note that the somatosensory feedback controller has been removed due to the focus on auditory feedback in these theories. Adapted from Guenther (2016).

To aid comparison of these different sensorimotor accounts of stuttering, Figure 4 illustrates the major sites of disruption proposed by each account projected onto the DIVA model framework. It can be seen that the different theories propose disruptions to a wide variety of areas within sensorimotor control of speech. It should be kept in mind when viewing this figure that not all of these theories use the DIVA model as their framework for speech motor control, and so mapping their sites of disruption onto this model is not always straightforward. In particular, the *Unstable Internal Models* hypothesis does not assume the presence of inverse models in the feedback control system, and so this connection in the DIVA model is not marked with a red cross for this theory.

A major question that differentiates these theoretical perspectives concerns whether a change in auditory feedback processing reflects a primary site of disruption in stuttering, or a secondary downstream consequence of disruption to a different neural system. The *SMS* account and the *Disrupted Auditory Prediction* account both consider dysfluencies to be the result of the impaired functioning of one of the two control subsystems in isolation; that is, the faulty operation of the feedforward or feedback control system directly causes stuttered speech. In contrast, other accounts propose that it is the interactions between the feedback and feedforward control systems that result in disruptions to fluent speech. For example, in the *Overreliance on Feedback hypothesis*, even though impairments in feedforward control lead to increased articulatory errors, these by themselves are not a major impediment to fluency if speech is controlled solely by the feedforward control system; crucially, it is the operation of the feedback controller on these speech errors that results in dysfluencies. Similarly, in the *Impaired Left Hemisphere Basal Ganglia Motor Loop* account, disruption to the readout of feedforward motor commands leads to the detection of sensory errors in feedback that prevent the proper generation of initiation and termination signals by the basal ganglia. In this way, while auditory feedback processing plays a role in all of the reviewed theories of stuttering, the theories differ in whether feedback processing is specifically impaired (i.e., the primary source of the problem), or simply a secondary consequence of some other disruption.

There are several other divisions that can be made between these theories that flow from this central distinction. The first concerns whether recruitment of feedback control in speech is seen as compensatory or maladaptive. In the *SMS* and *Unstable Internal Models* accounts, slowing of speech in order to enhance processing of sensory feedback is seen as a strategy employed to help speech motor control. Conversely, although the *Overreliance on Feedback* hypothesis also predicts increased use of sensory feedback in speech, this is itself a cause of dysfluencies, due to the accumulation of sensorimotor error. Feedback control of speech is similarly seen as maladaptive in the accounts proposed by Guenther (2016) and Max and Daliri (2019).

A further related distinction that can be made between theories concerns whether changes in feedback control are seen prior to stuttering onset, or whether these develop with experience of stuttering. The *Unstable Internal Models* hypothesis, *Overreliance on Feedback* hypothesis, and the *Disrupted Auditory Prediction* account all propose changes to auditory feedback control processes or their relative weighting in speech motor control as part of the original pathology in stuttering; these theories thus predict that differences in feedback processing should be seen from the onset of the disorder. Conversely, the *SMS* and *Impaired Left Hemisphere Basal Ganglia Motor Loop* accounts both propose that changes to feedback processing develop with experience of stuttering, suggesting a compensatory increase or decrease in reliance on sensory feedback over time respectively. Therefore, these accounts would predict that changes in sensitivity to auditory feedback should only be seen in individuals with significant experience of stuttering, and not from stuttering onset.

#### Predictions for performance on altered feedback paradigms

The different assumptions made by these theories have implications for their predictions regarding the compensation and adaptation responses of PWS during perturbations of auditory feedback. A summary of these predictions along with other key features of these accounts are presented in Table 1. It can be seen that altered feedback paradigms thus have the potential for testing the predictions of these theories, in order to start evaluating which provide the best fit to observed behaviour in stuttering.

**Table 1.** Summary of theories and their predictions

**Theory**	**Feedforward control disrupted**	**Feedback control disrupted**	**Feedback control compensatory or maladaptive**	**Changes in feedback control developmental or acquired after onset**	**Predicts impairment in compensation for unexpected feedback perturbations**	**Predicts impairment in adaptation to sustained feedback perturbations**
**Speech Motor Skills account** (van Lieshout et al., 1996a, 1996b)	Yes	No	Compensatory	Acquired	No (may be enhanced in adults)	Yes?
**Unstable Internal Models hypothesis** (Max et al., 2004)	Yes	Yes	Compensatory	Developmental	Yes	Yes
**Overreliance on Feedback hypothesis** (Civier et al., 2010; Max et al., 2004)	Yes	No	Maladaptive	Developmental	No (may be enhanced)	Yes
**Impaired Left Hemisphere Basal Ganglia Motor Loop account** (Chang & Guenther, 2020; Guenther, 2016)	Yes	No	Maladaptive	Acquired	Yes (weakened in adults only)	Yes (possibly more affected in adults)
**Disrupted Auditory Prediction account** (Max & Daliri, 2019)	No	Yes	Maladaptive	Developmental	Yes	Yes?

Impaired compensation for unexpected perturbations of auditory feedback would be predicted by theories that assume disruption within the feedback control system. The *Unstable Internal Models* account would predict that disruption to forward models should result in an impaired ability to detect and correct for sensory errors online, resulting in weaker or more variable compensation responses. The *Disrupted Auditory Prediction* account would similarly predict that a failure to properly prime the auditory system for processing of upcoming speech feedback would result in impaired detection of auditory errors, and thus weaker compensation responses. The *Impaired Left Hemisphere Basal Ganglia* account would also predict weaker compensation responses to unexpected feedback perturbations, but only in adults or individuals with significant experience of stuttering who have learned to reduce reliance on sensory feedback as a compensatory strategy. Conversely, children who stutter should not show differences in compensation responses relative to children who do not stutter. In contrast to these theories, the *Overreliance on Feedback* hypothesis does not assume any disruption within the feedback system itself, and so would predict intact or possibly even enhanced compensation responses in both children and adults who stutter.

Conversely, impaired adaptation to sustained perturbations would be predicted by theories that propose malfunctioning within the feedforward control system for speech. The *Unstable Internal Models* account would predict disruption to the adaptation response, due to problems in the updating of stored inverse models within the feedforward system. The *Overreliance on Feedback* hypothesis and *Impaired Left Hemisphere Basal Ganglia Motor Loop* account both propose disruption within the feedforward system, and so would predict abnormalities of the adaptation response. In both theories, however, it is the readout of feedforward commands that is affected, and not the updating of stored inverse models on which sensorimotor learning relies. It is therefore unclear exactly how the adaptation response would be affected, and whether there would be no learning at all. However, Chang and Guenther’s (2020) suggestion of a decreased reliance on sensory feedback over time would presumably also affect the adaptation response; that is, sensory errors would not be incorporated into stored inverse models, leaving feedforward commands unchanged to result in no learning. This account would thus be compatible with a difference in the adaptation response between children and adults who stutter.

The *Disrupted Auditory Prediction* account does not assume impairments in the feedforward system, and the authors suggest that the PSAM effect may have greater functional relevance for online corrections of speech gestures via the feedback control system (Max & Daliri, 2019). However, it is difficult to see how offline updating of internal models within the feedforward system could remain unaffected by disruption to the auditory system’s ability to detect sensory errors. The DIVA model explicitly assumes that such updating of feedforward models underlying the adaptation response relies on detection of errors by the feedback system (see dashed arrows in Figure 2). While there is evidence that the adaptation response can be selectively impaired without disruption to compensation responses (e.g., Parrell et al., 2017), to our knowledge there is currently no evidence that a successful adaptation response can occur in the absence of an intact compensation response. Disrupted auditory prediction would therefore likely result in an impairment in the adaptation response.

The predictions made by the *SMS* hypothesis regarding differences in compensation and adaptation responses in PWS are less clear, since this theory is not rooted in a sensorimotor control framework as explicitly as the other theories. However, on the premise that this view is consistent with an impairment in automated feedforward speech motor control that triggers increased reliance on sensory feedback, we can assume this theory would predict weakened or more variable adaptation responses but enhanced compensation responses in PWS.

It can therefore be seen that these theories make different predictions regarding patterns of behaviour of PWS in feedback perturbation studies. In the last decade, several studies have begun investigating compensation and adaptation responses in PWS. Reviewing this body of evidence can thus start to provide insight into which of the theoretical accounts best explains the pattern of behaviour of PWS in such experiments. The following section will therefore present the evidence to date using altered feedback paradigms with PWS, highlighting where findings support particular theories over others.

### Review of Evidence from Altered Feedback Experiments

#### Pitch shift studies

Early studies of compensation responses to pitch perturbations in PWS reported small effects, often in underpowered samples. Bauer et al. (2007) reported a trend for PWS to show slower compensation responses to unexpected pitch shifts than PWNS, although in a small sample of just four PWS. Specifically, when averaging the response across trials within-subjects, PWS showed longer mean latencies and delayed response peak times. Loucks et al. (2012) reported similar effects in a larger sample (*n* = 14), with PWS showing significantly delayed average onset latencies in their compensation responses. This study also reported weaker average compensation responses in PWS, although this was based on qualitative description of trends, and not statistically significant differences. A more recent study by Sares et al. (2018) was able to demonstrate a statistically significant reduction in the average response (area under the curve) to pitch shifts in a sample of 19 PWS; however, further analyses were presented to challenge a straightforward interpretation of this effect. They argued that the weaker averaged compensation response in PWS was attributable to (1) the decreased number of opposing trials (versus following trials or trials with no compensation) shown in PWS, and (2) the greater variability in the timing of their opposing responses. Crucially, when looking at only those trials on which an opposing response was made, no significant group difference in the magnitude of compensation was found between PWS and PWNS.

These results suggest that the feedback control system of PWS is able to detect and appropriately compensate for auditory errors online, but the timing of the implementation of such corrective commands involves increased variability. This could mean that on some trials, there is not sufficient time for the delayed response to reach its peak magnitude, resulting in an overall reduced compensation magnitude when averaged across trials. In a recent fMRI study, Sares et al. (2020) were not able to replicate the group difference in the average compensation response when the paradigm was conducted in the scanner, despite using a subset of the same participants from their previous study. They attributed this to aspects of the in-scanner environment such as the external noise; however, it could also simply highlight the unreliability of this effect.

Despite this lack of behavioural differences, Sares et al. (2020) did nevertheless find differences in underlying neural activation between PWS and control participants who did not stutter. PWS were found to show deactivation of the middle temporal gyrus (MTG) and superior frontal gyrus (SFG) in the right hemisphere for shifted versus unshifted trials; conversely, PWNS showed increased activity in these areas for shifted versus unshifted trials. The authors proposed that these two areas may form a processing circuit in which the MTG informs the SFG about auditory feedback, which in turn engages a compensatory response. Furthermore, independent components analysis identified differences in the functional networks engaged in the two groups; while controls demonstrated engagement of a network that integrated both auditory and motor components, auditory and motor networks were dissociated in PWS during vocalisation. This suggests weaker communication or coordination between auditory and motor regions, which could underlie the increased variability in timing of the compensation response.

Theoretically, this increased variability in timing of the compensatory response perhaps best fits the *Unstable Internal Models* hypothesis. Instability in forward and inverse models within the feedback control system could mean that while in some cases appropriate corrective commands can be generated, instability of modelling at other points results in a failure to initiate the compensation response sufficiently quickly. Such instability in the formation of internal models could be underwritten by the weakened communication between auditory and motor regions reported by Sares et al. (2020). Similarly, this would fit with the account proposed by Hickok et al. (2011), which suggests that noisy/variable forward predictions result from disruption to area Spt which coordinates the mapping between motor and sensory areas.

#### Formant perturbation studies

##### Unexpected perturbations.

Studies of compensation responses to unexpected formant perturbations have also reported some contradictory findings. A study by Cai et al. (2012) found reduced compensation responses to unexpected perturbations of F1 in 21 PWS. In further analyses, it was demonstrated that these reduced responses were not attributable to (1) increased variability in responses across trials, (2) delayed response latencies, or (3) impaired auditory acuity for perceiving differences in the F1 of vowels. Furthermore, the proportion of compensating trials did not differ between groups. This pattern of results thus differs from that reported by Sares et al. (2018) with unexpected pitch shifts, in that weaker responses could not be attributed to differences in timing of the responses.

Conversely, a further study from this same group failed to find evidence of weaker compensation to unexpected formant perturbations. Cai et al. (2014) investigated compensation to unexpected F2 perturbations during production of the multisyllabic phrase “I owe you a yo-yo.” In this task, PWS were found to demonstrate normal compensatory responses that looked similar to those of controls. Although the authors described a trend for these responses to have a slower onset in PWS, this was not manifest in any significant group differences. This discrepancy in findings between Cai et al. (2012) and Cai et al. (2014) could be explained by multiple methodological differences between the studies, such as the nature of the perturbation (a sudden step-like increase in F1 versus a gradual ramp up in F2) and the target utterance (single words versus a multisyllabic utterance).

In a second experiment reported by Cai et al. (2014), however, a significant group difference was found in response to a temporal perturbation. This involved random acceleration or deceleration of auditory feedback (specifically, advancing or delaying the timing of the perceived F2 transition). Interestingly, controls failed to demonstrate compensation for either type of perturbation; instead they showed following responses on deceleration trials only, in which they further lengthened syllable productions. PWS, on the other hand, lacked any significant timing adjustments in response to the temporal perturbations. The authors interpreted this as evidence that stuttering involves slowness in auditory-motor integration for temporal control. However, interpretation of this behaviour is made difficult given that the response of controls was not to compensate for the temporary perturbation. Instead, their so-called “following responses” suggest that timing of speech motor control was disrupted by the deceleration of feedback. Conversely, the speech of PWS appeared to be resistant to the disruptive effects of this auditory feedback perturbation. This pattern echoes the effects of delayed auditory feedback on speech motor control, resulting in dysfluent and disrupted speech in controls but improved fluency in PWS.

The lack of compensation for these temporal perturbations in controls makes it difficult to relate this behaviour to an error-based correction mechanism within the feedback control system. Instead, this perturbation is more likely to disrupt the proper generation of timing cues by the basal ganglia as outlined in the GODIVA account (Bohland et al., 2010); that is, since auditory feedback is effectively delayed, the generation of a termination and initiation cue for the next phoneme will be delayed also, resulting in a slowing of speech. The behaviour of PWS in this study thus fits with Guenther’s (2016) proposal that a deficit in the use of auditory feedback to guide generation of internal timing cues leads to a reduced reliance on auditory feedback. Thus, the timing of speech motor control in PWS is less affected by temporal perturbations of auditory feedback. In this way, this difference in response of PWS to this type of auditory feedback perturbation may more likely reflect compensatory changes in sensitivity to auditory feedback, rather than a central point of disruption.

It is interesting to note that when citing these two studies by Cai and colleagues, authors often appear to present their findings as converging evidence for weaker compensation responses in PWS. As is apparent from the current review, however, the findings from these two studies in fact demonstrate quite different forms of disruption to sensorimotor responses to altered feedback in PWS and, in the case of compensation to unexpected perturbations of F2, report conflicting results. It will therefore be important for future literature to ensure the findings from these studies are accurately represented when making claims about compensation responses in PWS.

##### Sustained perturbations.

Preliminary suggestions that adaptation responses may be abnormal in PWS were found in a serendipitous finding reported by Cai et al. (2012). Although their paradigm involved unexpected perturbations, a cross-trial adaptation effect was found in control participants, in which an early “following” response was seen in perturbed trials that were immediately preceded by a perturbed trial of the opposite direction (e.g., a downward shift trial preceded by an upward shift trial). This was interpreted as evidence that some offline updating of internal models had occurred in response to that previous perturbation. Interestingly, such cross-trial adaptation was entirely absent in PWS, suggesting problems in the updating of stored sensorimotor models.

Accordingly, subsequent studies reported weaker adaptation responses in PWS in sustained perturbation paradigms. Sengupta et al. (2016) reported reduced adaptation in response to a sustained perturbation of F1 and F2 in a sample of eight PWS participants. Similar results were found by Daliri et al. (2018), who reported significantly reduced adaptation in a group of 14 adults who stutter compared to controls. Interestingly, this was not found for a group of children who stutter, who showed significant adaptation to the same extent as control children. The authors therefore argued that abnormalities in the speech adaptation response in adult PWS were likely the result of a compensatory strategy learned over many years of stuttering, rather than the result of a primary deficit that contributes to stuttering onset. That is, experience of stuttering throughout childhood and into adulthood may lead to a reduced reliance on auditory feedback for speech motor control.

However, recent work by Kim et al. (2020) provides contradictory evidence to this previous study. Crucially, significantly weaker adaptation to a formant perturbation was seen in both adults and children who stutter compared to non-stuttering aged matched controls. Indeed, in this study, the difference in extent of adaptation between stuttering and non-stuttering participants was even greater in the child sample compared to the adult sample. This discrepancy in findings from Daliri et al. (2018) may reflect the type of formant perturbation employed; while Daliri et al. perturbed F1 and F2 in opposite directions so as to induce a shift in the phonemic category of the vowel sound, Kim et al. employed a global formant perturbation in which all formants were perturbed. This results in a change in the perception of the size of the vocal tract that produced the vowel. Kim et al. highlighted how sensitivity to this type of perturbation of auditory feedback would be highly relevant for adapting to the rapid maturational changes in the biomechanics of the vocal apparatus that occur during childhood; a failure to update one’s internal models to reflect these developmental changes in vocal anatomy would be problematic for accurate sensorimotor control during speech. It should be highlighted also that while Daliri et al.’s study included children aged 6 to 11 years, Kim et al. included children as young as three years (ranging up to 9 years). Indeed, in this study the most severe impairments in adaptation were seen in the youngest children who stutter, with adaptation improving with age in this group. They therefore concluded that impaired speech adaptation is not something that develops with experience of stuttering, but is in fact present very early in development, close to the onset of the disorder. This is consistent with the view that this disruption to the adaptation response may be causally relevant for the disorder.

As well as comparing adults and children who stutter, this study by Kim et al. (2020) also compared adaptation responses within-subjects in response to formant perturbations that were either introduced gradually in a ramp-like fashion, or suddenly (i.e., the maximum perturbation is employed in a single-step). In the group of adults who stutter, a greater reduction in adaptation compared to controls was found in the sudden perturbation condition compared to the gradual perturbation condition. Indeed, significant adaptation at the group level was seen in the adults who stutter in response to a gradual upward (but not downward) perturbation (although this was still significantly reduced in magnitude compared to PWNS). Thus, a gradual ramping up of the formant perturbation appeared to aide adaptation in adults who stutter. This was, however, not the case in children who stutter, who failed to show significant adaptation for either the gradual or sudden perturbation condition. The authors speculated that this difference in adults may reflect differential impairment of cortical, basal ganglian, and cerebellar circuits, with some evidence that these structures may be differentially recruited for these different types of perturbation (Criscimagna-Hemminger et al., 2010; Robertson & Miall, 1999; Venkatakrishnan et al., 2011).

A study by Kim and Max (2020) aimed to further investigate the reasons for reduced adaptation in PWS. Firstly, they replicated this finding in an F1 perturbation paradigm with a greater number of perturbation trials (120 trials) than had been used in previous studies; adaptation in PWS thus does not seem to benefit from extended exposure to altered feedback. Secondly, they investigated the potential contribution of an explicit component to speech adaptation in PWS and PWNS. In visuomotor adaptation for reaching movements, participants are known to employ explicit aiming strategies in order to combat large sensory errors caused by sudden perturbations, in addition to implicit updating of internal models (Bond & Taylor, 2015). In their study, Kim and Max (2020) found that neither group reported explicit awareness of intentions to change their speech when repeatedly asked after each trial (with the exception of one PWNS participant). This accords with other evidence suggesting that speech adaptation to formant perturbations indeed only involves an implicit component (Lametti et al., 2020; Munhall et al., 2009). Finally, this study also looked at whether participants’ perceptual targets for the spoken words were changed during the task, given evidence that speech motor learning can induce changes in the perception of speech sounds (Lametti et al., 2014; Shiller et al., 2009). It is possible that reduced adaptation in PWS could be due to greater shifts in their perceptual targets in the direction of the perturbation, reducing perceived sensory error and thus adaptation. Participants were asked to select an acoustic stimulus that best represented each of the test words at repeated time-points throughout the task; however, neither group demonstrated any change in the F1 of their selected targets after adaptation. Overall, therefore, these authors concluded that reduced adaptation in PWS reflects disruption to implicit sensorimotor learning that relies on the updating of internal models.

In order to further understand the nature of the disruption to adaptation responses in PWS, several studies have employed EEG measures in concert with speech motor learning paradigms. Previous work with PWNS has reported changes in phase coherence in the theta-band during speech adaptation, which were attributed to the formation of a new feedforward map with learning (Sengupta & Nasir, 2015). Building on these findings, and alongside their reporting of behavioural group differences, Sengupta et al. (2016) compared phase coherence changes during speech adaptation between PWS and PWNS, and found widespread group differences across various frequency bands. Specifically, PWS showed abnormalities in alpha coherence early in the perturbation phase (higher alpha-beta and alpha-gamma coherence than controls), whereas abnormalities in theta coherence were seen late in the perturbation phase (theta-beta coherence increased over adaptation in PWNS but not in PWS). Based on the timing of these differences, alpha coherence abnormalities early in adaptation were suggested to reflect abnormal processing of feedback error, while theta coherence abnormalities late in adaptation were suggested to reflect abnormal learning of a new feedforward map. They therefore concluded that their behavioural findings of weakened adaptation responses in PWS (as discussed previously) may reflect aberrant communication within sensorimotor networks that affects both the detection of sensory errors and the updating of internal feedforward models.

EEG measures were also related to adaptation responses in PWS by Daliri and Max (2018), who investigated the potential relationship with PSAM (see section, Disrupted auditory prediction and feedback monitoring). As previously reviewed, the PSAM effect has been reported to be absent in PWS (Daliri & Max, 2015a, 2015b); this raises the possibility that reduced adaptation responses in PWS could be caused by a failure to appropriately prime the auditory system to process sensory errors in auditory feedback (reflected in reduced PSAM). Although they were able to replicate both reduced speech adaptation and reduced PSAM in a sample of 13 PWS, surprisingly a negative correlation between the magnitude of these effects was found in the PWS group, with no significant relationship at all in the control group. That is, PWS who showed greater PSAM showed reduced speech adaptation. This is not consistent with a view in which PSAM enhances sensitivity of the auditory system to sensory errors, and is difficult to interpret given that both PSAM and adaptation were weaker in PWS. Therefore, although these altered responses at a group level in PWS both support the notion of disrupted sensorimotor integration, how they may relate to one another remains unclear.

Overall, findings of weaker adaptation responses in PWS are consistent with a number of theories that propose disruption to feedforward control in stuttering (Chang & Guenther, 2020; Civier et al., 2010; Max et al., 2004), or to auditory prediction that would affect updating of internal models in the feedforward system (Max & Daliri, 2019). Evidence from Kim et al. (2020) demonstrates that this disruption can be seen early in childhood, near to the onset of the disorder. They related this evidence to Max et al.’s (2004) *Unstable Internal Models* account, arguing that a failure to learn accurate or stable internal models would seriously hamper the speech sensorimotor system’s ability to flexibly adjust to maturational changes in the vocal apparatus that occur throughout childhood. Their global formant perturbation was specifically selected to mirror this type of maturational change by inducing a change in perception of the size of the vocal tract (of course over a much shorter timescale than in development itself). However, Daliri et al.’s (2018) finding of intact adaptation in children (but not adults) who stutter in response to a formant perturbation that affected the identity of a vowel sound suggests that some aspects of auditory-motor learning for speech may be relatively unaffected in childhood, instead becoming progressively worse with experience of stuttering into adulthood. This is perhaps more consistent with the *Impaired Left Hemisphere Basal Ganglia Motor Loop* account (Chang & Guenther, 2020; Guenther, 2016). Although this proposes a core deficit in the left hemisphere feedforward system, it also acknowledges that the use of auditory feedback in guiding speech motor control may change over time with experience of stuttering, as PWS learn to suppress auditory feedback as a compensatory strategy. Why responding to these two types of formant perturbations and their corresponding auditory feedback errors should be differentially affected in children who stutter, however, cannot be readily explained by any current theories.

## DISCUSSION

This review of evidence from altered feedback studies has found that there are differences in the way in which PWS use auditory feedback to guide speech production. Compensation responses to unexpected perturbations appear to be disrupted; however, this evidence is not always consistent and may reflect issues with the timing of the response rather than a straightforward weakening. This is not always adequately conveyed when these articles are cited in the literature; future studies citing this work should ensure that it is interpreted correctly. Adaptation responses to sustained perturbations appear to be consistently weaker across studies in adults who stutter, as well as in children who stutter when certain formant perturbations are used. Overall, therefore, PWS appear to be less effective in the use of feedback to guide their production of speech sounds, across both unexpected and sustained perturbation paradigms.

### Evaluating Theories of the Use of Feedback in Stuttering

The overall pattern of evidence speaks directly against the *Overreliance on Feedback* hypothesis, despite this being a highly influential theory in the field. Instead, this theory appears to fit much better with the pattern of behaviour shown by Parkinson’s patients in altered feedback experiments. Specifically, compensation for unexpected perturbations is enhanced (Chen et al., 2013; Huang et al., 2016; Liu et al., 2012), whereas adaptation to sustained perturbations is reduced (Abur et al., 2018; Mollaei et al., 2013). This clear dissociation between responses in the two paradigms fits with the idea of a core disruption to feedforward control causing an overreliance on feedback control. Parkinson’s disease is a limb motor control disorder caused by degeneration of dopaminergic neurons in the substantia nigra (pars compacta) of the basal ganglia. Interestingly, a number of speech disturbances including stuttering-like dysfluencies are often reported in patients (Juste et al., 2018). It is possible therefore that an overreliance on feedback control may contribute to stuttering-like behaviour in some cases.

Contrasting this evidence from Parkinson’s patients with the currently reviewed findings from PWS highlights how the pattern is much more complex in developmental stuttering. This suggests that a simple account of impairment in one control system and overreliance on the other is not sufficient in this case. This likely reflects the fact that stuttering is a developmental and not an acquired disorder. That is, it is important to recognise that the impairments to speech motor control seen in developmental stuttering are not the result of damage to the mature system. Surprisingly, this point is apparently often overlooked. For example, the DIVA model simulations presented by Civier et al. (2010) involved inducing a shift in balance between feedback and feedforward control *after* learning in the model was complete. As such, this does not provide a viable model of a developmental disorder. It is vital that any theory of sensory feedback disruption in stuttering considers the importance of dynamic interactions between different parts of the speech motor control system as it develops.

Evidence from studies of sustained feedback perturbations demonstrate that the adaptation response is disrupted (weaker) in adults who stutter. Furthermore, this disruption can be seen early in childhood and thus potentially contributes to the cause of the disorder (Kim et al., 2020). However, there is also evidence that the severity of this impairment may change across development. Kim et al. (2020) reported an improvement in the adaptation response to a global formant perturbation with increasing age in children who stutter, with the worst impairment seen in an early age group. Conversely, the evidence from Daliri et al. (2018) suggests that adaptation to a formant perturbation that affects the identity of a vowel gets worse over time in a person who stutters, remaining intact in childhood but becoming impaired by adulthood. It is difficult to know how to reconcile these two findings. Kim et al. (2020, p. 11) argued that the *Unstable Internal Models* hypothesis does not assume that children who stutter would not be able to “correct their productions when auditory feedback from previous trials indicates that they produced the words with a completely wrong sound.” However, it is hard to imagine how children who stutter would be able to show any kind of speech adaptation if their core deficit was in the feedforward system and internal models.

Overall, the evidence reviewed here reveals abnormalities of both compensation and adaptation responses in PWS. This suggests that the disruption to sensorimotor learning processes in stuttering has repercussions for the operation of both the feedback and the feedforward control systems. This is consistent with the *Unstable Internal Models* hypothesis (Max et al., 2004), in which there is a failure to appropriately update both inverse models in the feedforward system and forward models in the feedback system. This latter disruption to forward models that facilitate prediction of the sensory consequences of a speech motor gesture would also be consistent with the *Disrupted Auditory Prediction* account (Max & Daliri, 2019). A failure to accurately predict the sensory consequences of speech movements would result in erroneous sensory error signals, affecting both online compensation responses to unexpected feedback perturbations, and consequently the appropriate updating of inverse models in the feedforward system when perturbations are sustained. More work is needed, however, to better understand how these disruptions may change over time, and how this may manifest differently in response to different types of errors in auditory feedback. In particular, these accounts will need to reconcile the finding of intact adaptation in children who stutter in response to perturbations that affect perceived vowel identity (Daliri et al., 2018). This finding itself would benefit from replication in a sample including younger-aged children, in order to determine whether such an adaptation response remains intact at an age closer to typical onset of stuttering.

### Role of Development and Potential Heterogeneity

This discussion highlights the difficulty of interpreting disrupted responses to altered feedback measured in the mature system. These responses reflect the end point of an abnormal developmental trajectory, and as such represent the combined result of both primary disruptions and their secondary consequences, including compensatory strategies. In this way, it can be difficult to tease apart these different aspects when relying on evidence from a single point in time. Longitudinal studies of responses to altered feedback in children who stutter, including comparisons between those who go on to recover and those whose stuttering persists, will likely provide better insight into how dynamic interactions between different subparts of the system may be critical for development of fluent speech.

It is also important to consider that the causes of speech motor control problems in developmental stuttering may be heterogeneous across the population. That is, stuttering may emerge when there is a disruption to the balance across subsystems, but the exact pattern of this disruption may vary across individuals. This could explain some of the inconsistencies in the reviewed evidence, such as the failure of all studies to find overall weakened responses to unexpected perturbations of auditory feedback in adult PWS.

### Comparing Auditory and Somatosensory Feedback Control

It will be important for future theorising on the role of sensory feedback in stuttering to integrate evidence across different modalities. The current review focused on theories and evidence considering the role of auditory feedback in stuttered speech, because this has been most extensively studied in altered feedback and sensorimotor learning paradigms. However, there is a separate literature considering the role played by somatosensory feedback in stuttering (Archibald & De Nil, 1999; Loucks & De Nil, 2006). PWS have been found to show reduced accuracy and increased variability in jaw movements, exacerbated by an absence of visual feedback and increased time-pressure (Loucks & De Nil, 2006). There is also some evidence that their motor control system is less able to take jaw size into account during movement planning (Daliri et al., 2013).

However, to our knowledge there are currently no studies of sensorimotor learning with somatosensory feedback perturbations in PWS. Where manipulations of somatosensory feedback have been used with PWS, these have typically involved alterations such as tendon vibration, equivalent to masking of auditory feedback. In order to consider disruptions to the use of auditory and somatosensory feedback within a common framework, it would be of interest to investigate the ability of PWS to compensate for jaw perturbations in paradigms comparable to those reviewed here in the auditory domain (e.g., Tremblay et al., 2003). That is, are the compensation and adaptation responses shown by PWS during somatosensory perturbations similar to those seen in the auditory domain?

This would be of interest to questions concerning the development of stuttering, given that somatosensory feedback is thought to play a less important role in early typical speech development (Tourville & Guenther, 2011; Trudeau-Fisette et al., 2019). Indeed, it has been shown that individuals can vary in their relative weighting of these two sources of feedback during speech motor control (Lametti et al., 2012). It would be of interest to consider whether the same is true in PWS, or whether as a group they are more likely to use one particular form of feedback control over the other. In the DIVA model, distinct auditory and somatosensory feedback controllers are proposed, which would allow for their selective disruption; alternatively, it is possible that a more general disruption (e.g., to internal modelling) may affect the operation of both feedback controllers in a similar way. More work is therefore needed to compare auditory-based and somatosensory-based feedback control in PWS.

### Summary and Conclusions

This review has sought to provide insights into the role of sensory feedback in developmental stuttering, by bringing together evidence on responses of PWS in altered auditory feedback paradigms. This evidence mostly favours a theoretical perspective that proposes a disruption to the updating and use of internal models in speech motor control in stuttering, affecting both feedback and feedforward control of speech. Overall, this field of research would benefit from an improved focus on the developmental aspects of the disorder, and consideration of interactions between feedback processing across auditory and somatosensory modalities.

## FUNDING INFORMATION

Carolyn McGettigan, Leverhulme Trust (https://dx.doi.org/10.13039/501100000275), Award ID: RL-2016-013.

## AUTHOR CONTRIBUTIONS

**Abigail R. Bradshaw**: Conceptualization: Lead; Investigation: Lead; Writing – original draft: Lead; Writing – review & editing: Lead. **Daniel Lametti**: Conceptualization: Equal; Supervision: Equal; Writing – review & editing: Equal. **Carolyn McGettigan**: Conceptualization: Equal; Funding acquisition: Lead; Supervision: Equal; Writing – review & editing: Equal.

## TECHNICAL TERMS

Feedback perturbations: Refer to experimentally induced changes in the real-time sensory feedback a speaker receives during speech production.
Feedback control: A mode of motor control in which sensory feedback is used to control a movement online.
Feedforward control: A mode of motor control involving pre-planned motor commands that are not corrected by sensory feedback online.
Compensation responses: The changes a speaker makes to an ongoing vocalisation to counteract an unexpected perturbation of sensory feedback.
Adaptation responses: Correspond to the gradual changes made across repeated speech productions to counteract a sustained perturbation of sensory feedback.
Forward models: Used in motor control to translate motor commands into their predicted or desired sensory consequences.
Inverse models: Used in motor control to translate sensory outcomes into the motor commands needed to achieve them.
